# Preserving Source Location Privacy for Energy Harvesting WSNs

**DOI:** 10.3390/s17040724

**Published:** 2017-03-30

**Authors:** Changqin Huang, Ming Ma, Yuxin Liu, Anfeng Liu

**Affiliations:** 1 School of Information Technology in Education, South China Normal University, Guangzhou 510631, China; cqhuang@zju.edu.cn; 2 School of Information Science and Engineering, Central South University, Changsha 410083, China; minma@cs.stonybrook.edu (M.M.); afengliu@mail.csu.edu.cn (A.L.); 3 Department of Computer Science, Stony Brook University, New York, NY 11794, USA

**Keywords:** energy harvesting wireless sensor network, preserving source-location privacy, redundancy branch convergent routing, network lifetime

## Abstract

Fog (From cOre to edGe) computing employs a huge number of wireless embedded devices to enable end users with anywhere-anytime-to-anything connectivity. Due to their operating nature, wireless sensor nodes often work unattended, and hence are exposed to a variety of attacks. Preserving source-location privacy plays a key role in some wireless sensor network (WSN) applications. In this paper, a redundancy branch convergence-based preserved source location privacy scheme (RBCPSLP) is proposed for energy harvesting sensor networks, with the following advantages: numerous routing branches are created in non-hotspot areas with abundant energy, and those routing branches can merge into a few routing paths before they reach the hotspot areas. The generation time, the duration of routing, and the number of routing branches are then decided independently based on the amount of energy obtained, so as to maximize network energy utilization, greatly enhance privacy protection, and provide long network lifetimes. Theoretical analysis and experimental results show that the RBCPSLP scheme allows a several-fold improvement of the network energy utilization as well as the source location privacy preservation, while maximizing network lifetimes.

## 1. Introduction

Fog (From cOre to edGe) computing, a term coined by Cisco in 2012, is a distributed computing paradigm [1], that empowers network devices at different hierarchical levels with various degrees of computational and storage capability [1,2,3,4]. According to [1], since 2011 the number of connected devices has already exceeded the number of people on Earth. The connected devices have reached nine billion and are expected to grow more rapidly and reach 24 billion by 2020 [1,5,6]. With this increasing number of heterogeneous devices connected to the IoT and the generating data [4,7,8,9,10,11], the security and privacy of such devices are becoming an urgent issue [4,6,12,13,14,15]. In particular wireless embedded devices such as smart mobile devices and sensors are an increasingly ubiquitous technology used during the daily lives of people worldwide [16,17,18,19,20]. Ensuring the security and privacy of embedded devices is a challenging issue since such devices generally operate with a limited power energy budget provided by batteries or solar cells [21,22], must often work in a potentially hostile environment and offer limited hardware support for resisting attacks due to cost constraints, so they are vulnerable to various attacks, including attacks in terms of security (such as on-off attacks, fault injection attacks, memory-based attacks and other attacks [4,12,13,14,15,23,24]), and attacks related to privacy preservation (such as leakage of source-location privacy, and inference of private information [25,26,27,28,29,30,31,32,33,34]). According to statistics, there are more than 30 types of attack in sensor networks [35], with source-location privacy attacks being a simple attack behavior which can cause great harm to the network [23,27,33,34].

Energy Harvesting Wireless Sensor Networks (EHWSNs) [21,22] are networks which can harvest energy from the surrounding environment (such as solar energy, wind energy, thermal energy, vibration, etc.) to supplement the node energy. By replenishing devices with renewable energy, EHWSNs can be deployed in harsh environments for a long time or perform permanently unattended monitoring. They are also called the green networks [21,22,36,37,38], since the energy used is renewable, without causing much inference or damage to the surrounding environment. Thus, EHWSNs have attracted extensive attention from researchers [21,22,39]. Security is one of most important factors in the development and applications of sensor networks [13,23,25,27,29,31,40,41]. One crucial security issue is the preservation of source location privacy [25,26,27,28,29,30,31,32,33,34]. In wireless sensor networks (WSNs), a source node can detect an event at the event source and transmit the data to the sink. The event source can be a soldier lurking in the battlefield, an armed policeman performing a special task, or a rare protected wild animal (e.g., a panda) [25,31]. If the adversary can detect the source location by wireless location tracking technology, it could pose a severe threat to the soldiers, armed policemen or the animals in the forest reserve [25,26,27,28,29,30,31,32,33,34].

Numerous research works related to source-location privacy preservation have been proposed. To the best of our knowledge, however, most existing methods are not aimed at providing source-location privacy preservation for EHWSNs. In traditional WSNs, one of most important research topics is how to save energy and prolong the network lifetime [42,43,44,45], whereas in EHWSNs, sensors can collect energy from the surrounding environment (e.g., solar energy, wind energy, and other green energy). Even if a sensor become inoperative temporarily because it runs out of energy, the sensor can recover and work normally when the environment allows for energy collection again [21,22,39]. However, energy harvesting in EHWSNs is not stable, suffering from restrictions in various aspects. For instance, the speed of energy collection in a sensor is related to the weather and seasonal conditions [21,22,39]. Therefore, energy efficiency is a complicated problem in EHWSNs.

Despite of existence of numerous research works in source-location privacy preservation [25,26,27,28,29,30,31,32,33,34], there still exists a large scope for further exploration. We summarize the novelties of our work presented in this paper as follows:

(1) The proposed privacy-preserving scheme is equipped with a strong privacy preserving ability and remarkable resistance to global attacks. In this paper, we propose a redundancy branch convergence-based preserved source location privacy (RBCPSLP) scheme for EHWSNs. In the RBCPSLP scheme, data generation is not mainly triggered by an event, but by the amount of remaining energy. On the whole, when each node sends data it is an independent event, without any regularity. Even if armed with a global view, an adversary cannot determine where the true event source is, since most of the generated event sources are fake ones, thus we do not mention the case of an adversary with a local view.
(2) The proposed privacy-preserving scheme enables the network to achieve high lifetime performance. In WSNs, the sensor nodes near the sink consume a great deal of energy and may die early, thus resulting in the energy hole phenomenon [2,5,23], whereas the sensor nodes far from the sink consume relatively less energy and have surplus energy. As indicated in some studies, up to 90% of the energy can remain unused when a network dies due to the impact of energy holes. Based on the above observations, therefore, we propose a novel redundancy branch convergence-based preserved source location privacy (RBCPSLP) scheme. Unlike existing privacy-preserving schemes, the data generated from each event source (including true event sources and fake ones) in the proposed RBCPSLP scheme is not independently transmitted to the sink. Instead, the data in route to the sink converge onto a single or a few routes. In contrast to the existing privacy preserving schemes in which each fake route transmits data to the sink independently, the RBCPSLP scheme reduces the energy consumption in hotspot areas significantly, and thus enhances the network lifetime.

The rest of this paper is organized as follows: in Section 2, related works are reviewed. The system model is described in Section 3. In Section 4, a novel redundancy branch convergence-based preserved source location privacy (RBCPSLP) scheme is presented. Performance analyses are provided in Section 5. Section 6 presents our experimental results and a comparison with literature methods. We conclude in Section 7.

## 2. Related Work

The goal of a privacy-preserving scheme is to conceal the location information of source nodes, while sending the generated data from the source node to the sink, thus it becomes a dilemma [25,28]. In order to forward the generated data packets from the source node to the sink, there must be a route from the source node to the sink, but it is not hard for an adversary to locate the source node using backtracking. In theory, therefore, there always exists the probability of being attacked. Numerous methods have been proposed to address this problem, which are summarized as follows:

(1) Data flooding schemes. These have the following characteristics: regardless of whether an event occurs or not, each node generates data periodically or fills packets with useless data, and then transmits data packets with the same length to the sink using the same routing strategy. In such a scheme [34], the adversary cannot determine the location of the true event source since there is no distinction between all the nodes from the perspective of an adversary, thus the privacy of the source location is well preserved. This privacy-preserving scheme can not only be applied to planar networks [25,28], but also to cluster routing networks [29]. However, the disadvantage of this flooding scheme lies in the fact a huge burden is imposed on the system and this reduces the network lifetime if all the sensor nodes generate data, regardless of whether the event occurs or not. An alternative scheme employing the random selection of sensor nodes for data transmission was then proposed in [46] to alleviate the problem. On the whole, however, the schemes in this category are hard to apply in practical applications due to their large negative influence on the network lifetime.
(2) Phantom routing. Because of the production of a large amount of useless data, flooding privacy-preserving schemes dramatically lower the network lifetime. Thus, some new schemes only allow the event source nodes to generate data, and transmit this data based on a single-path routing scheme to the sink. However, the traditional single-path routing schemes (e.g., shortest path routing, SPR) are vulnerable to attacks using backtracking to trace the location of source nodes. Thus, some improved routing schemes are proposed for the privacy-preserving single-path routing. One such important scheme is the phantom routing scheme proposed by Kamat et al. in [30], which consists of two stages: (1) a data packet goes through ϒ-hops to a phantom source node; (2) the data packet is then transmitted from the phantom source node to the sink via a flooding scheme or shortest path routing. The first stage of the routing process is aimed at generating a phantom source node that is far from the true source node with diverse location possibilities, making it difficult for an adversary to track down to the true source node. The second stage is targeted at the transmission of the data packet to the sink. The advantage of this scheme is that only one routing path need be used to transmit the data to the sink, which can save a great deal of energy compared to the flooding strategy, resulting in a higher network lifetime. In the phantom routing scheme, it is critical to generate phantom nodes that are far away from the source node. The generation of the phantom source node was achieved by random routing in earlier research [20]. Subsequent research found that in the random routing scheme, the phantom nodes actually had less than a ϒ/5 distance from the source node after ϒ‑hops. The closer the phantom node is to the source node, the worse its security is.

However, there are some deficiencies in the existing research: (1) in the studies based on phantom routing, the improvement of phantom routing is mainly focused on how to keep the phantom nodes away from the source nodes, thus increasing the difficulty for the adversary to find the source node. Generally, the phantom node is not far from the source node, so the adversary can still find the source node by using other combined methods, so the security cannot be guaranteed; (2) in most studies, the routing from the phantom node is based on the shortest routing algorithm, and its route is similar to the line connecting the phantom node and the sink. Thus, an adversay can easily trace the phantom node in this direction, and then follow the phantom nodes to locate the source node. Therefore, there are many studies on the improvement of phantom routing strategies [27,28].

According to the adversary’s attack strength, source location privacy-preserving schemes can be divided into two categories as follows: (1) those able to resist a global attack. Global attacks refer to those in which the adversary has a global view allowing it to monitor the wireless signals of the entire network, and then use a variety of methods to infer the source location [34]; (2) those able to resist local attacks. Local attacks refers to those in which the adversary only has a 1-hop field of view, that is, the adversary can only detect the wireless signala in a range of one hop, so the attack can only be carried out by hop-by-hop backtracking. Obviously, an adversary with a global view has a very large destructive power, and therefore is extremely difficult to resist [31,33]. The methods used to resist global attacks are based on flooding schemes, so they are extremely energy-intensive. Fortunately, it is hardfor an adversary to achieve general status, because it requires remarkably expensive equipment, and the use of conspicuous equipment might expose the adversary easily. Thus, in practice, attacks most often come from an adversary with a local view [31].

In terms of the object which needs privacy preservation, there is a scheme to achieve the privacy preservation of the sink location. Its goal is to prevent the adversary from knowing the location of the sink. Generally, the false sink method is employed for privacy preservation of the sink location, that is, one selects a certain number of nodes in the network as a sink, then each node will not only send its own data to the true sink, but also send it to the false sink to confuse the adversary [34]. The main disadvantage of this scheme is that each node needs to send data to n sinks. This results in n times the amount of data, seriously affecting the network lifetime. In [47], another privacy-preserving scheme was proposed, which connects all the sinks together. Each node sends data to only one sink, but the data are exchanged between the sinks. In [48], a mobile sink privacy-preserving scheme was also proposed.

## 3. The System Model and Problem Statement

### 3.1. The Network and Adversary Model

#### 3.1.1. Network Model

In this paper, the wireless sensor network model is as follows:

(1) The network studied in this paper is a sensor network with a radius R. The sensor nodes are evenly distributed in the network. The nodes cannot be moved after their deployment. The density is ρ. Once an event occurs, the nodes near the event will generate the data and transmit data to sink via multi-hop routing [2,8,10,25,28].
(2) Suppose the monitored targets are randomly distributed in the network. That is, the probabilities of detecting the target for each sensor node are equal, and the probabilities of reaching sink for the data are also equal [2,8,10,25,28].
(3) This paper assumes that the network has the basic security facilities. For example, the secure communication protocol between the nodes has been established, and the communication between nodes uses secure encryption communication. Therefore, all the information in the network is not known to the external environment. The methods for secure encryption and key management are beyond the scope of this paper. For details of these methods, readers are referred to [6,18].

#### 3.1.2. Adversary Model

This paper assumes that the adversary has a very strong attack capability. Due to the great importance of the attack, the adversary is often equipped with advanced equipment [16,20,21]. The adversary’s initial state is at the sink, observing the communication between the sink and its neighbor nodes. Once a node sends a data packet to the sink, the adversary moves quickly to a node that sends data and then monitors the next data packet. This process continues until it cannot detect any data packets, or find the true source node [25,28,29,30]. In the RBCPSLP scheme proposed in this paper, we also extend the condition that the adversary only has a local field of view. We prove that even if the adversary has a global view, the RBCPSLP scheme still has a good privacy-preserving capability. The adversary model in this paper has the following properties:

(1) The adversary has infinite energy, that is to say, the adversary’s energy consumption is not considered [25,28,30]. Also, the adversary’s storage capacity is large enough. Once an adversary detects the presence of a data packet, it can immediately determine the location of the sending node by analysing the strength and direction of the signal. Once the sender’s location is determined, the adversary can move to the sender’s location instantly. Moreover, the adversary will not miss any data packet that is within the adversary’s monitoring range [25,28,30].
(2) The adversary can only perform passive tracking. It can neither interfere with the normal routing functions in the network, nor tamper with the data and damage the sensor devices since such aggressive disruptions are likely to expose the adversary. Therefore, the adversary only performs passive data snooping [25,28,30].

### 3.2. Energy Consumption Model and Related Definitions

**Figure 1:**
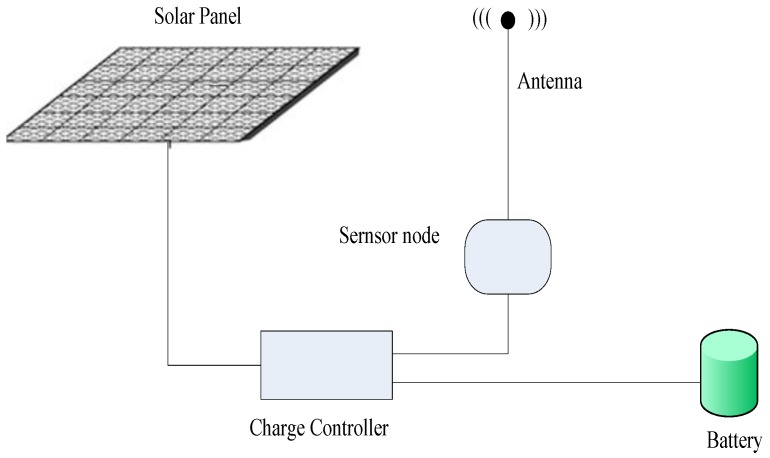
Solar node structure diagram.

In this paper, we use the most effective and most readily available solar energy harvesting wireless sensor network, with the structure shown in Figure 1, as the energy consumption model [39]. In such a model, a wireless sensor node usually consists of a processor module, a sensor module, a wireless communication module, a solar collector, and a battery [21,22,39], wherein the solar collector is an energy harvesting module, which plays a role of converting light into electrical energy by the photoelectric effect or the photochemical effect. Nowadays, thin film solar cells are used as the mainstream technique to convert light energy into electric energy using the principle of photoelectric conversion, and then stores the electrical energy in the battery [21,22,39]. The battery provides the energy for the energy consumption module of the sensor node. The power controller adjusts the transmission frequency of the wireless communication module according to the energy level of the battery, the sunshine time, the sunshine intensity, the day-night relationship and so on, so as to change the energy consumption condition of the wireless sensor nodes to make maximum use of the limited electric energy.

**Figure 2:**
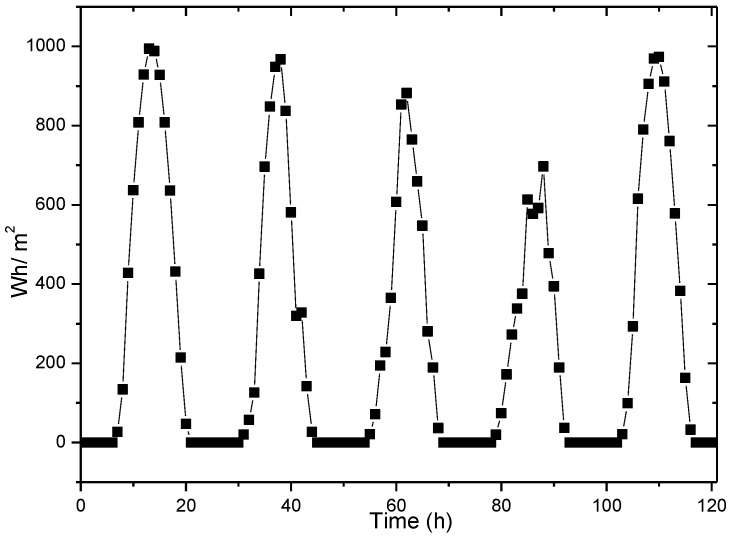
The amount of energy harvesting changes with time.

Since the solar energy is affected by the sun, its energy collection is periodic. The data in this paper are based on the radiation at Los Angeles (33° N, 118° W) from 1 August to 5 August 1990, as shown in Figure 2 [39]. It records the light information for every hour in a day. The light information is directly related to the intensity of energy harvesting, which actually reflects the energy harvesting rate.

This paper adopts the typical energy consumption model [2,8,25,28,49]. The energy consumption of sending data is shown in Equation (1), while the energy consumption of receiving data is expressed in Equation (2):

(1) $$\{\begin{array}{ll} E_{t}=lE_{elec}+l\varepsilon_{fs}d^{2} & \mathrm{if}\;d<d_{0}\\ E_{t}=lE_{elec}+l\varepsilon_{amp}d^{4} & \mathrm{if}\;d>d_{0} \end{array}$$

(2) $$E_{r}(l)=lE_{elec}$$

where $E_{elec}$ denotes the energy consumption of the transmission circuit. If the transmission distance is less than the threshold $d_{0}$, the power amplification loss adopts the free space model. If the transmission distance is greater than or equal to the threshold $d_{0}$, the multipath fading model is adopted. $\varepsilon_{fs}$ and $\varepsilon_{amp}$ represent the energy required for power amplification in the abovementioned two models, respectively. l is the number of bits of data. In this paper, the specific settings of the above parameters are same as those in [25], as shown in Table 1.

### 3.3. Problem Statement

The problems in this paper can be summarized as the following three aspects:

(1) The proposed scheme enables good privacy preservation for source nodes. In general studies, the privacy preservation is evaluated according to the number of hops ϒ that an adversary k needs to backtrack to the source node t. $h_{i,j}$ refers to the hop counts from node i to node j. The larger ϒ is, the greater the cost is for the adversary to trace the source node. Thus, the objective of the privacy-preserving scheme is expressed as the following Equation (3):
  (3) $$\max(ϒ)=\max(\sum_{i=t}^{k}h_{t,i})$$
(2) Maximize the network lifetime. The energy of a sensor node in the WSN is very limited. Therefore, the goal of this paper is to maximize the network lifetime. In this paper, the network lifetime refers to the first node death time (FDT). Suppose the energy consumption of a node i is $e_{i}$. The maximization of the network lifetime is to minimize the energy consumption of the node with the maximum energy consumption:
  (4) $$\max(T)=\min\;\underset{0<i\leq n}{\max}(e_{i})$$
(3) Maximize the use of energy. That is, take full advantage of the energy collected by the network to create more interferences signal or routing paths to enhance privacy preservation. Numerous studies have pointed out that, in energy harvesting WSNs, the focus of this type of network is not the energy conservation, but how to make full use of the available energy. Ideally, all energy collected in the network are consumed. This is the so-called energy neutrality [50]. The amount of energy collected is dynamic. It is desirable to ensure the battery energy is not watsed (i.e., the energy collection rate is not greater than energy consumption rate when the battery capacity is full). Meanwhile, the remaining energy of the node cannot be less than the lowest threshold. Suppose an energy harvesting period T is divided into n smaller time slots τ. $E_{con}^{j}$ denotes the energy consumption in the j-th time slot, then the maximization of energy use is expressed by the following Equation (5):
  (5) $$\max\left(E\right)=\max\left(\sum_{j=0}^{n-1}E_{con}^{j}\right)$$
  To sum up, the goal of this paper is expressed as follows:
  (6) $$\left\{ \begin{array}{l} \max(ϒ)=\max(\sum_{i=t}^{k}h_{t,i})\\ \max(T)=\min\;\underset{0<i\leq n}{\max}(e_{i})\\ \max\left(E\right)=\max\left(\sum_{j=0}^{n-1}E_{con}^{j}\right) \end{array}\right.$$

## 4. The Design of the Privacy-Preserving Scheme

### 4.1. Design of the RBCPSLP Scheme

**Figure 3:**
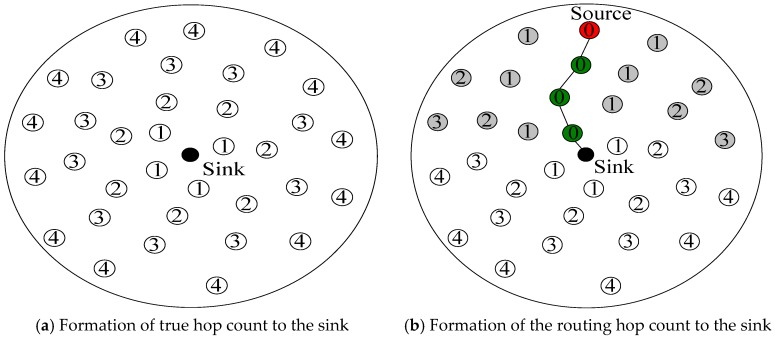
Formation of routing distance.

In this paper, we propose a distributed privacy-preserving strategy. Figure 3 shows the formation of the RBCPSLP scheme, which consists of the following stages:

(1) The stage in which each node obtains the true hop count used to arrive at the sink.

At the beginning of the RBCPSLP scheme, the minimum hop count to the sink is formed for each node. This process is described as follows: at the time of initialization, the sink sets its own hop count to 0 (i.e., $v_{0}.h_{1}=0$ where $v_{0}$ denotes the sink and its attribute $h_{1}$ represents the true number of hops used to reach the sink), and all the other nodes set their own hops to reach the sink to infinity. The sink broadcasts that its hop count to the sink is zero. Suppose the information field containing the hop count in the data packet B is $h_{m}$. Then, the node receiving the data packet (e.g., node $v_{i}$) updates its hop count to the sink according to the following comparison:

(7) $$v_{i}.h_{1}<B.h_{m}+1$$

If the above formula holds, the node $v_{i}$ updates its hop count to sink according to the following Equation (8):

(8) $$v_{i}.h_{1}=B.h_{m}+1$$

Otherwise, the node $v_{i}$ does not take any action. If the node $v_{i}$ updates its own hop count to reach the sink, then it broadcasts its hop count in the broadcast packet. The above process repeats until the hop counts of all nodes are not updated or a predetermined update time threshold is reached. The formation process is shown in Figure 3a.

(2) The stage in which the routing hop count of each node is formed.

The routing hop count to the sink refers to the number of hops with which a node routes the data to sink. In the RBCPSLP scheme, the node uses the shortest path route (SPR) algorithm to route the data to the sink, that is, each node selects the node closest to the sink as the next hop. Although the SPR algorithm is adopted, each node in the RBCPSLP scheme does not choose the true nearest node to the sink as the next hop, but rather selects the routing hop count defined in this paper. The routing hop count of a node $v_{i}$ to the sink is represented by $v_{i}.h_{2}$, and it is determined as in [49].

After the hop count of each node to reach the sink is determined, we set the routing hop count of each node to the sink as $v_{i}.h_{2}=v_{i}.h_{1}$. In the subsequent routing process, each node selects the shortest routing hop count to route data to the sink. However, unlike previous routing schemes, each node will set its routing hop count to 0 (i.e., $v_{i}.h_{2}=0$) after it selects its shortest routing path. Then each node updates $h_{2}$ with a similar approach to obtaining $h_{1}$.

The process of routing to the sink is shown in Figure 3b. When the source node generates data, it uses the shortest routing algorithm to send data to the sink according to the routing hop count to the sink, forming a routing path as shown in Figure 3b. Then, the routing hop count in the routing path is set to 0 and the routing hop count to the sink is broadcasted. Affected nodes near the routing path update their routing hop counts to reach the sink. As shown in Figure 3b, the gray nodes indicate that their routing hop counts to the sink have been updated.

(3) Routing stage.

In previous privacy-preserving routing schemes, the number of fake source nodes cannot be too large in order to reduce the energy consumption in the hotspots area near the sink, so the number of source nodes is determined by the upper bound of the number of data s undertaken by the hotspot area. If the hotspot area is able to take m data packets in an energy collection cycle, each node can last for α time slots for routing, and the cycle length of sending data packet is τ (i.e., the length of the time slot), then the number of the source nodes that can be selected is represented by the following Equation (9):

(9) $$\upsilon =\frac{m}{t/\tau }=\frac{m\tau }{t}$$

In the RBCPSLP scheme, multiple routes are merged into one route. Each route does not forward data directly to the next node after receiving the data, since there is only one or a few real source data in the network. Instead, it only needs to send the real source data to the sink, and it is not necessary to send all other fake data to the sink, therefore, each node waits for a fixed cycle before forwarding data. In other words, a route to the sink in a cycle sends only one data packet (for multiple real source data, they are fused into one data packet using data fusion technology). Therefore, in the RBCPSLP scheme, the selected fake event source data does not have a one-to-one correspondence with the number of routes to the sink. Multiple fake source nodes correspond to one route to the sink, so the energy consumption of its hotspot area is only 1/k of the energy consumption of the existing schemes. Thus the network lifetime is significantly improved.

**Figure 4:**
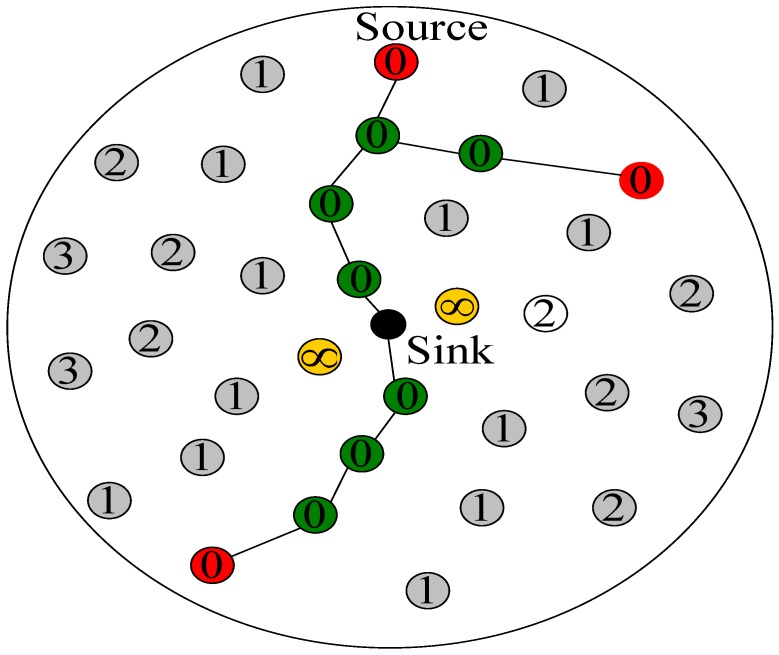
Redundancy branch converging routing scheme.

In this paper, we further optimize the RBCPSLP scheme according to the following method: when a node has k routes to the sink, it selects k nodes in its one-hop range (i.e., the hotspot area) to forward the data. This node sets its hop count to the sink as 1, while other nodes in its one-hop range change their distance to the sink to infinity. In this way, only k nodes in the one-hop range broadcast their distance to the sink is 1, and at most k converging routes to the sink are formed using the algorithm similar to the aforementioned stage one. Finally, the routing scheme of the RBCPSLP is shown in Figure 4. In the figure, ∞ indicates the node’s routing hop count to the sink is infinity, without the task of forwarding data. The pseudo code for the routing algorithm of the RBCPSLP scheme is shown in algorithm 1.

**Algorithm 1. RBCPSLP routing****Stage 1: get the real hop to Sink for each node** **1**: Set each node $v_{i}.h_{1}=\infty$ //set its hop count to Sink as ∞ **2**: Sink sends a broadcast with the value of HotptoSink = 0; //Sink starts to broadcast its hop count to Sink is 0, i.e., $B.h_{1}=0$ **3**: **For** each node $v_{i}$ which receive broadcast B
**Do** **4**:  **If**
$\upsilon_{i}.h_{1}<B.h_{1}+1$*< then* 5:   $\upsilon_{i}.h_{1}=B.h_{1}+1$; **6**:   broadcast with $B.h_{1}=\upsilon_{i}.h_{1}$
**7**:  **Else** **8**:    waiting **9**:  **End If** **10**: **End For** **11**: **Stage 2 and 3: routing to Sink** **12**: **For** each node $v_{i}$
**Do** **13**:  $v_{i}.h_{2}=v_{i}.h_{1}$ **14**: **End For** **15**: **For** each node $v_{i}\notin S_{v}$//$S_{v}$ denotes the set of nodes in its one-hop range **16**:   $v_{i}.h_{2}=\infty$ **17**: **End For** **18**: **For** each node which $v_{i}.h=1$ and $v_{i}\notin S_{v}$ **19**: **For** each node $v_{i}$ which receive broadcast B or packet **Do** **20**:  **If** receive packet *then*
**21**:   select next node u using Algorithm 3 **22**:   send packets to u **23**:    Set $v_{i}.h_{2}=0$ **24**:    Broadcast B
**with**
$v_{i}.h_{2}=0$ **25**:  **Else** **26**:   **If**
$\upsilon_{i}.h_{2}<B.h_{2}+1$
*then* **27**:   $\upsilon_{i}.h_{2}=B.h_{2}+1$; **28**:   broadcast with $B.h_{2}=\upsilon_{i}.h_{2}$ **29**:   **End If** **30**:   **End If** **31**: **End For**

### 4.2. The Time Slot When the Source Data is Generated and Its Generation Method

Although the RBCPSLP scheme is given, there are still some important issues which have not been addressed yet. The first important issue of the RBCPSLP scheme to be addressed in this section is how to generate as many branch routes as possible, that is, fake source nodes. When are they generated? How long does it take? How are they evenly distributed in the whole network? More importantly, how can each maintain the original energy after the completion of an energy harvesting cycle so that the network lifetime can last forever (or the lifetime be maximized) since the number of branch routes is determined by the energy. Supposes the cycle of energy harvesting is T (it is 24 h for the solar energy), and T can be divided into smaller slots with the same length (e.g., τ). Let $E_{hrv}^{j}$ denote the energy collected during the slot j, $E_{con}^{j}$ the energy consumption during the slot j. Therefore, the remaining energy of the battery in the time slot k can be calculated by the following Equation [49]:

(10) $$E_{btr}(t_{0}+k\tau )=E_{btr}(t_{0})+\sum_{j=0}^{k-1}\left(E_{hrv}^{j}-E_{con}^{j}\right)$$

where $E_{btr}(t)$ represents the remaining energy of the battery in the time slot t, and $t_{0}$ denotes the starting time slot 0. Therefore, the goal is to find the energy consumption in a series of time slots:

$$E_{con}=\langle E_{con}^{0},E_{con}^{1},...,E_{con}^{n-1}\rangle$$

such that the total energy consumption E is maximized, that is, the network energy utilization rate is maximized:

(11) $$\left\{ \begin{array}{l} \max\left(E\right)=\max\left(\sum_{j=0}^{n-1}E_{con}^{j}\right),\;s.t\\ E_{btr}(t_{0}+k\tau )=E_{btr}(t_{0})+\sum_{j=0}^{k-1}\left(E_{hrv}^{j}-E_{con}^{j}\right),\forall 0\leq k\leq n\\ 0\leq E_{btr}(t_{0}+k\tau )\leq E_{btr}^{\max},\;\forall \;0\leq k\leq n,\\ 0\leq E_{con}^{k}\leq E_{btr}(t_{0}+k\tau ),\;\forall \;0\leq k\leq n,\\ 0\leq E_{con}^{k}\leq E_{con}^{\max},\;\forall \;0\leq k\leq n \end{array}\right.$$

where n=⌈T/τ⌉ is the number of time slots in the time cycle T. $E_{btr}^{\max}$ denotes the maximum capacity of the battery, $E_{con}^{\max}$ represents the maximum allowable energy consumption in a slot. As the battery capacity is limited, the battery cannot be replenished if it reaches maximum capacity while charging. When the battery’s remaining energy is below a certain threshold (such as 0), energy cannot be consumed. Also, $E_{con}^{k}$ cannot be greater than $E_{btr}(t_{0}+k\tau )$. Therefore, the above Equation (11) is a constrained optimization problem. 

In the RBCPSLP scheme, each node generates its own data independently, and routes data independently to the sink according to the routing algorithm proposed in the previous section. Numerous routes will be finally converged into a few number of routes converging to the sink. Each node is required to randomly select a time slot to send data with a fixed cycle τ as the source node. As a source node, it sends a data packet in each cycle τ. Therefore, the energy consumed in a time slot τ is also set to fixed ϖ (even if a node acting as the source node forwards data of other nodes, it only consumes energy ϖ in each cycle, so the energy consumption of forwarding data of other nodes is not considered for the node working as a source node). In this way, we set a node to choose a random time slot, and starts to send data from this slot. Then the maximum number α of cycles allowed to send data from this beginning of this slot is the solution to the problem. Assuming that the random selections starts from the i-th slot, and lasts for α slots, then the problem of the energy assignment is converted into the following Equation (12):

(12) $$\left\{ \begin{array}{l} \max\left(\alpha \right),\;where\;E_{con}=\langle E_{con}^{i},E_{con}^{i+1},...,\left(E_{con}^{i+\alpha }|E_{con}^{n}\right)\rangle ,\;s.t\\ E_{btr}(t_{0}+k\tau )=E_{btr}(t_{0}+i.\tau )+\sum_{j=i}^{n}\left(\bar{E_{hrv}^{j}}-E_{con}^{j}\right),\forall 0\leq k\leq n\\ 0\leq E_{btr}(t_{0}+k\tau )\leq E_{btr}^{\max},\;\forall \;i\leq k\leq n,\\ 0\leq E_{con}^{k}\leq E_{btr}(t_{0}+k\tau ),\;\forall \;i\leq k\leq n,\\ E_{con}^{k}=\varpi ,\;\forall \;i\leq k\leq i+\alpha ,\\ E_{con}^{k}=\omega ,\;\forall \;k\geq i+\alpha ,\;E_{k}^{n}=E_{k-1}^{n} \end{array}\right.$$

where the energy consumption in each slot is the fixed ϖ once each node starts to act as the source node in the period (i⋅⋅⋅i+ατ). ω is the energy consumption in the period (i+ατ+1, nτ), and such energy consumption is used for forwarding data. The condition $E_{k}^{n}=E_{k-1}^{n}$ means that the energy of node’s battery is equal to the energy at the end of the previous energy harvesting cycle T, i.e., neutral energy consumption.

We use the pre-estimated method to solve the problem in the above Equation (12). The idea is that we first predict the energy harvesting in the cycle and then calculate the maximum number of cycles α satisfying the condition in above Equation (12). We first introduce the prediction method for energy harvesting. 

The energy harvesting power is related to the first few cycles of energy harvesting. Therefore, in this paper, we first use the information in the first few cycles of energy harvesting to predict the current cycle of energy harvesting. 

**Figure 5:**
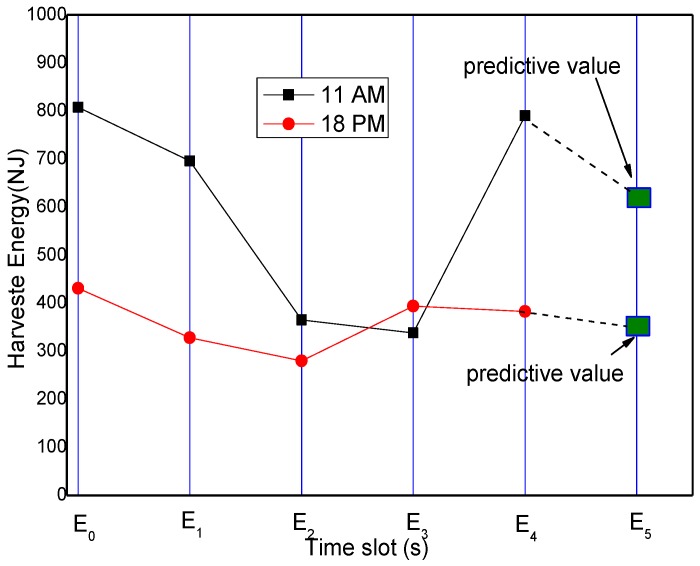
Predicted harvesting energy based on previous rounds.

From the energy harvesting situation given in Figure 2, it can be seen that the energy harvesting cycle is 24 h for the solar energy collector. Between 0 a.m. and 7 a.m. in each cycle, the energy harvesting rate is very low or even zero, while reaches its maximum at noon. Likewise, we divide each energy harvesting cycle into n smaller slots. Let $E_{hrv}^{k,i}$ represent the amount of energy collected in the i-th slot of the first k energy harvesting cycles away from the current prediction cycle. This paper uses basic historical information estimation method (see Figure 5) for prediction. For instance, the data from the first w energy harvesting cycles are used to predict the amount of energy collected in current cycle, as expressed in the following Equation (13):

(13) $$\bar{E_{hrv}^{i,1}}=\sum_{k=1}^{w}\left(E_{hrv}^{k,i}.\varphi (k)\right)/\left(w-\left(\frac{\left(w-1\right)}{2}\right)\right)$$

where φ(k)∈[0, 1] is the attenuation function, which is used for a reasonable weighting in the estimation of the energy harvesting amount at different times. According to the characteristics of energy harvesting, the energy harvesting amount in the current slot is closely related to the energy harvesting in the recent energy harvesting slots. Since the amount of harvested energy in summer is large due to intense light for a long period of time, while it goes down to a very low level in the winter, we use the recent period of energy collection to predict the amount of harvested energy in some future time slot. We estimate the amount of energy collected in a future slot by the amount of energy collected in recent w slots. Obviously, the closer to the current slot, the greater the weight of that slot. Therefore, we adopt attenuation function with weighted coefficients defined as Equation (14):

(14) $$\varphi (k)=\{\begin{array}{c} 1,\\ \varphi (k-1)=\varphi (k)-1/w, \end{array}\begin{array}{c} k=w\\ 1\leq k<w \end{array}$$

**Figure 6:**
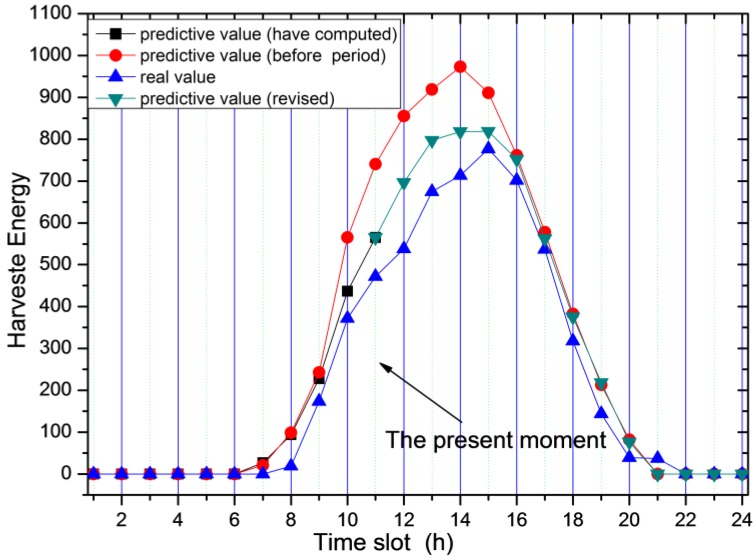
Predicted value vs. the actual value of the energy.

The curve of the dotted line in Figure 6 indicates the predicted value calculated according to Equation (13). The curve with reverted triangle marks represents the actual value. It is observed that there is a certain difference between the predicted value and the actual value. The reason for this difference is that solar collection is closely related to the weather conditions, and therefore is closely related to the previous cycles in terms of the magnitude of the predicted values, but more related to the current cycle of energy harvesting.

**Figure 7:**
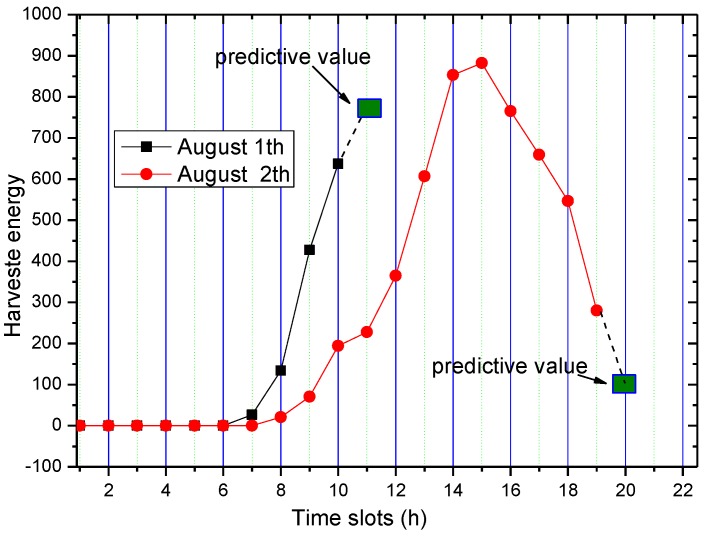
Predicted amount of energy harvesting based on the current value.

Therefore, we propose a method to modify Equation (13) in real time according to the actual situation of the current cycle. As shown in Figure 7, although the previous prediction method can predict the energy harvesting rate of the current cycle, there still exists a large difference between the predicted values and the actual situation.

In Figure 7, the plot for predictive value just shows the trend of the predicted value of energy, so the predicted value after 11 h is not plotted in the figure. It can be obtained that as time increases, the predicted value increases as well. Therefore, we also use the actual energy harvesting rate of current to predict the energy consumption of remaining period of the cycle. The method used in this paper is the three-point method which predicts energy harvesting rate of the next time slot τ. Suppose the actual energy harvesting rate of current slot k is $E_{hrv}^{k}$, and the energy harvesting rates of two recent slots k − 1 and k − 2 are $E_{hrv}^{k-1}$, $E_{hrv}^{k-2}$ respectively. Then we let:

(15) $$\Delta_{k}=E_{hrv}^{k}-E_{hrv}^{k-1},\;\Delta_{k-1}=E_{hrv}^{k-1}-E_{hrv}^{k-2}\;\Delta =(\Delta_{k}+\Delta_{k-1})/2$$

If $\Delta_{k}$ > 0, then the current energy harvesting ascends, so the energy harvesting rate of the next time slot is predicted to be

(16) $$E_{hrv}^{k+1}=E_{hrv}^{k}+\Delta$$

If $\Delta_{k}$ < 0, then the current energy harvesting descends, so the energy harvesting rate of the next time slot is predicted to be:

(17) $$E_{hrv}^{k+1}=E_{hrv}^{k}-\Delta$$

For the prediction method of energy harvesting rate in the remaining time slot, we adopt the correction method. Specifically, as shown in Figure 6, the dotted curve represents the predicted values according to Equation (13), the curve with black square marks denotes the actual values, and the curve with large green square is the predicted values for the remaining time slots. The correction method works in the following way: compare the predicted value calculated using Equation (13) with the actual value is compared, and then we compute their difference. Finally we add the corrected difference value to the predicted value:

(18) $$\Delta s=\sum_{i=1}^{k+1}\left(E_{hrv}^{i}-\bar{E_{hrv}^{i,1}}\right)/\left(k+1\right),\;E_{hrv}^{i,\Delta }=\bar{E_{hrv}^{i,1}}+\Delta s$$

After the energy harvesting rate of each remaining time slot is calculated, the node can independently compute the a. Here we use exhaustive search method. Suppose the current time slot is i, and we initialize a = 1. Then we calculate whether Equation (12) is satisfied. If so, let a=a+1, and repeat this process to increase a until Equation (12) cannot be satisfied or a + i ≥ ζ (ζ is the max value which is set by system). The algorithm for computing a is given below. Note that the node determines whether the data source is generated based on its own energy. When the energy is insufficient to support sending data the in the next time slot, the node will terminate the data transmission earlier. The following Algorithm 2 gives the prediction and correction of the energy harvested value and the calculation method of a slots for the node acting as the source node.

**Algorithm 2. Predict harvested energy and compute**
a**1**: **For** each $\tau_{i}\in T$
**Do**   //predict harvested energy for each slot in the energy harvesting cycle **2**: compute $\bar{E_{hrv}^{i,1}}$ using Equation (13) **3**: **End for** **4**: compute $E_{hrv}^{k+1}$ using Equation (18)   //predict harvested energy in k + 1 slot at the slot k **5**: **For**
i∈(1,k+1)
**Do**   //calculate the difference between the actual value and predicted value for   //each slot in (1, k + 1). **6**:  $s=s+E_{hrv}^{i}-\bar{E_{hrv}^{i,1}}$ **7**: **End For** **8**: $\Delta_{s}=s/\left(k+1\right)$ **9**: **For**
i∈(k+1, n)
**Do**   //correct the harvested energy from slot k + 1 to *the end of energy harvesting cycle* **10**:  $E_{hrv}^{i,\Delta }=\bar{E_{hrv}^{i,1}}+\Delta s$ **11**: **End For** **12**: **For** each node $v_{i}$
**Do** **13**:  i
**= random (1, n)**   //i is the slot when $v_{i}$ starts to act as data source. **14**:  a
**= 1** **15**:  **Do while** (a meets Equation (12) **16**:   a=a + 1 **17**:  **End Do** **18**:  a
**=**
a
**− 1** **19**: **End For**

### 4.3. Slot Competition and Selection of Next Hop

Each node randomly selects a time slot to send data, and the expected number of data packets to be sent is the α calculated in the previous Section. When the data transmission slot of the node ς arrives, the node ς needs to compete for slot. This is because only one node is needed to act as a data source node in the same communication interference area, and it is not necessary to generate multiple data sources in the same competition area. So when the node ς is ready to send data, it first broadcasts its own slot length and energy information to compete for the channel. At the same time, each node will monitor other nodes for their broadcast. The energy consumption $E_{hear}$ for overhearing is as follows:

In a time slot, suppose the communication interference radius is $r_{s}$, the number of information needed to be sent is ${r_{s}}^{2}\pi \rho \lambda$. So, the number of information that a node receives in a time slot is ${r_{s}}^{2}\pi \rho \lambda$. Since there are n time slots in a cycle, the number of information received by the node in a cycle is ${r_{s}}^{2}\pi \rho \lambda (n-\alpha )$. Thus, energy consumption for monitoring in a cycle is $E_{hear}={r_{s}}^{2}\pi \rho \lambda (n-\alpha )E_{elec}+{r_{s}}^{2}\pi \rho \lambda (n-\alpha )\varepsilon_{fs}{r_{s}}^{2}$.

When detecting that the remaining energy of some node is higher than its own remaining energy and meanwhile that node also starts to send data, the node will cancel its own data transmission slot and recalculate the new transmission slot (i.e., adding the originally planned slot and slots in the broadcasted data packet). If the node that plans to send data in the current slot finds that its remaining energy is greater than those of all other nodes that have broadcast their information, then the node broadcasts its own information. Finally, the winning node starts to send data.

In the RBCPSLP scheme, the probability that a node is selected as the source node is λ. In a time slot, when the node needs to send data, it first broadcasts information about its time slot and energy information in order to compete for the channel. In the meanwhile, each node monitors other nodes for their broadcast messages. It can be obtained that the broadcast information to be sent is $\lambda R^{2}\pi \rho$. When the broadcast information is propagated once, the number of nodes receiving the broadcast information is $r^{2}\pi \rho \lambda R^{2}\pi \rho$. When the node which has received the broadcast information starts to broadcast its own information, the number of nodes receiving its broadcast information is ${\left(2r\right)}^{2}\pi \rho \lambda R^{2}\pi \rho -r^{2}\pi \rho \lambda R^{2}\pi \rho$. Therefore, in the *k*-th broadcast of a node, the number of nodes receiving its broadcast information is ${\left(kr\right)}^{2}\pi \rho \lambda R^{2}\pi \rho -{\left((k-1)r\right)}^{2}\pi \rho \lambda R^{2}\pi \rho$. However, in general, at most one node is needed to act as the source node in the same communication interference area. Since each node has its own communication interference area, its total broadcast time slot and the number of data packets containing its own energy information is the number of all nodes in the entire network, i.e., $R^{2}\pi \rho$. Thus the total of amount of control packet is $R^{2}\pi \rho$.

Another problem is the selection of next-hop node in routing strategy. The following three aspects should be considered:

(1) The remaining energy of a node. Obviously, the more the residual energy of the node is, the greater the probability that the node is selected as the next hop is.
(2) The energy harvesting capacity of a node. A node in the sunshine has much greater energy harvesting rate than a node does under the shadow of an object. The node with strong energy harvesting capacity can collect a large amount of energy in a short time, while the node with weak energy harvesting capacity can hardly collect enough energy in a long time. Thus, the node with high energy harvesting rate should be selected as the next-hop node.
(3) Current data processing of a node. In the RBCPSLP scheme, each node in a state cycle selects a period of time to send data packets. Obviously, if there are two nodes with the same residual energy, and one node has completed its data transmission cycle while the other node has not started its data transmission, it is obvious that the node that has completed the data operation has a higher advantage as the next hop node because it does not need to consume energy in data transmission in the subsequent operation.

The following Algorithm 3 shows how to select the optimal next-hop node. Based on above mentioned three aspects, we use the weighted value of both residual energy and energy recovery rate as the selection criteria.

**Algorithm 3. Select optimization next node****1**: **For each**
$v_{k}\in N_{i}$  //for each node $v_{k}$ in the set of neighbors $N_{i}$ of node $v_{i}$ **2**: $\Lambda_{i}$ = $v_{i}.E_{hrv}^{j+1,\Delta }-v_{i}.E_{hrv}^{j}$  //$v_{i}.E_{hrv}^{j+1,\Delta }$ denotes the predicted value of node $v_{i}$ in slot j + 1 **3**: $e_{i}$ = $v_{i}.E$  //$v_{i}.E$ is the current energy of node $v_{i}$ **4**: $w_{i}=\varepsilon e_{i}+(1-\varepsilon )\Lambda_{i}$  //$w_{i}$ represents the weighted value of node $v_{i}$ **5**: **End For** **6**: **Return the node which with the max value**
$w_{i}$


### 4.4. Calculation of the Probability of the Data Generated by Nodes

At this point, we have discussed in detail the various aspects of the RBCPSLP scheme. However, an important parameter has not yet been determined, i.e., the energy consumption ϖ of data transmission in a slot of source data node as indicated in Equation (12). The energy consumption during the transmission period of non-source data node is ω, that is, the energy consumption of the node in forwarding the data from other nodes. ω is determined by the number of nodes acting as the data source. The more nodes that act as data sources, the greater the probability that a node will act as a relay node, and more data that will be transmitted. However, if more nodes act as data source, stronger source location privacy protection is needed, thus resulting in the damage of network lifetime. On average, the larger the number of source nodes is, the greater the energy consumption rate ϖ and ω of the nodes are. In a cycle of energy harvesting, the total energy that the network can obtain from the outside world is constant, and the obtained energy is not necessarily fully used. In sufficient sunshine, a node cannot continue to obtain outside energy due to full capacity of the battery. On the other hand, the energy obtained from outside world is dynamic, and related to different seasons and weather. Therefore, if a large number of source nodes are selected, the energy consumed by the network may be larger than that obtained from the outside world, so that the total energy of the network will decrease. If this trend continues, the energy of entire network will be gradually depleted, and thus bringing irreversible losses to the network. Therefore, how to balance the energy consumption of the network as well as the optimal relationship between the energy consumption and the intensity of privacy protection is another important aspect of the scheme.

For the purpose of ensuring the privacy of the source data, the main scheme proposed in this paper is to use the remaining energy to send multiple data as much as possible, making it difficult for an adversary to determine the real source data, so that the source data privacy is well preserved. In previous studies, it is known that when the node senses the data, the data will be transferred to the sink, resulting in high energy consumption of the nodes near the sink but low energy consumption of the nodes far from the sink. In this paper, the nodes use the solar panels to absorb energy. When the nodes far from the sink have relatively much residual energy, those nodes can make good use of the remaining energy to send data packets, and ensure he entire network lifetime. Nodes need energy to send data, when nodes have much remaining energy, they can send many data packets under the premise of not damaging the network lifetime. However, when the nodes have few remaining energy, they only send few packets or even do not send any data packets in order to ensure the network lifetime.

The RBCPSLP scheme proposed in this paper takes into account the factor that the total obtained energy network is fixed. Suppose the probability that a node is selected as a source node is λ, the network radius is R, and the node density is ρ, so the number of nodes in the network is $M=\pi R^{2}\rho$. The number of nodes acting as source data in an energy harvesting cycle is $M_{s}=\pi R^{2}\rho \lambda$. Since each node decides how many data packets it can send according to its own energy (the number of data packets actually corresponds to the number of slots in which the node acts the data source), it will send more data packets if there is sufficient energy, and send less data packets otherwise. The two parameters ϖ and ω are used in the computation of the number of data packets that a node can send when acting as a data source. ϖ refers to the energy consumption of sending a data packet, which is determined, so now the key is how to determine the parameter ω, which is related to the number of nodes that act as source nodes.

Each node predicts the total energy consumption that can be collected by the entire network during an energy harvesting cycle, based on the energy harvesting in the previous w energy harvesting cycles. Assume that the energy harvested by the node in the previous k-th energy harvesting cycle is $E_{hrv}^{k,en}$, then the predicted energy $\bar{E_{hrv}^{en}}$ that can be obtained in the current cycle is calculated in the following Equation (15):

(19) $$\bar{E_{hrv}^{en}}=\sum_{k=1}^{w}\left(E_{hrv}^{k,en}•\varphi (k)\right)/\left(w-\left(\frac{\left(w-1\right)}{2}\right)\right)$$

Thus the total energy obtained for the entire network is given by Equation (16) below, where φ is less than one, indicating the effective utilization rate of energy:

(20) $$\bar{E_{hrv}^{total}}=\varphi •M•\bar{E_{hrv}^{en}}$$

Suppose that in order to satisfy the security strength of source location privacy protection, g source nodes in each slot generate data. Then the energy consumed by the source nodes in a slot is gϖ. ω can be estimated by the number of hops used for each source data packet. We use f(ϖ) to represent the average number of hops that each source data needs to be forwarded, so the total energy consumption of the entire network for forwarding data packets is f(ϖ)gϖ. Generally speaking, the larger the ϖ is, the smaller the f(ϖ) is, and vice versa. Thus the total energy consumption in a slot is:

(21) $$\bar{E_{con}^{total}}=n•\left(f(\varpi )g\varpi +g\varpi \right)=n•g\varpi \left(f(\varpi )+1\right)$$

According to energy neutral rule, let:

(22) $$\bar{E_{con}^{total}}=E_{hrv}^{total}$$

That is:

(23) $$n•g\varpi \left(f(\varpi )+1\right)=M•\bar{E_{hrv}^{en}}$$

In the above formula, the maximum g can be found by exhaustive search. The probability that each node chooses itself as a source node is given by Equation (24):

(24) $$\lambda =\frac{g}{M}$$

From the abovementioned calculation process, we can see that the probability that the nodes become the source nodes is dynamically adjusted with the energy harvesting. When the solar energy is abundant in a season (e.g., in the summer), the probability of the node becoming the source node is higher; while when the solar energy is in a shortage season (e.g., in the winter), the harvested energy is reduced, so the the number of source nodes slected as data sources decreases, and the probability decreases as well. To the best of our knowledge, we have not seen a proposed similar scheme as ours with distributed feature, and full use of green energy, as well as with adaptive adjustment of the probability of sending data packets and maximum privacy protection capability. Therefore, this is an important nolvety of this paper.

In this paper if the energy prediction is not accurate, e.g., if the energy is under-predicted by 1/z, then we can obtain that the value of predicted energy is reduced. There is no sufficient energy for the nodes in the area far from the sink node to act as source nodes, and the number of data packets sent is also reduced by 1/z. When the energy is under-predicted by 1/z, the probability that each node chooses itself as a source node is decreased by 1/z. If all the nodes send data to the sink, the amount of data is reduced by 1/z as well, and the probability of an adversary to find the data source will be increased by 1/z. Although the energy prediction is not quite accurate, the security performance of data source is only reduced to (z − 1)/z. Therefore, it still increases the security of the network.

## 5. Performance Analysis of RBCPSLP Scheme

This section mainly analyzes the performances of the proposed RBCPSLP scheme in term of two aspects: network life and privacy preserving security.

### 5.1. Analysis of Network Lifetime

We first analyze the network lifetime. The following Theorem 1 holds for the network lifetime:

**Theorem** **1.** The RBCPSLP scheme can maximize the network lifetime.

**Proof.**  In the RBCPSLP routing strategy, each node acts as a source node with a certain probability. As can be seen from the analysis of Section 4, the node acts as the source data node according to the total amount of energy harvested by the network. If the prediction of the current energy harvesting cycle is accurate, the energy acquired in the current energy harvesting cycle can counteract the energy consumption of the nodes and thus does not reduce the energy reserve in the current energy harvesting cycle. Even if the prediction is not too accurate, according to the proposed RBCPSLP algorithm in Section 4, each node will decide whether to send data or not based on the amount of energy. If actual value of a node’s energy is lower than expected value, the node will not send the expected number of data packets. Thus the RBCPSLP scheme ensures that the energy of the node remains in the initial state of the energy harvesting cycle after an energy harvesting cycle is completed. Therefore, the RBCPSLP scheme can maximize network lifetime. ☐

### 5.2. Analysis of the Privacy Preserving Capability

(1) Analysis of traceback time.

Traceback time refers to the period from the time when the adversary starts to attack to the time when it tracks down the source node. In the attack mode with local vision, the adversary has the same sending radius with that of a sensor node. Therefore, the adversary can only detect the data packets in the one-hop range. After detecting the source of the data packet, we assume that the adversary has a very strong ability to move to the node that sends the data in no time. In such a case, the adversary can track the next hop node in r distance along the reversed routing path in τ time. Then in this case, it can be considered that the traceback time is proportional to the traceback distance (i.e., hops). Hence, we have the following Theorem 2.

**Theorem** **2.** *In the RBCPSLP scheme proposed in the paper, the average total routing path length is* $\Psi =\frac{\pi R^{2}\rho •\bar{E_{hrv}^{en}}}{n•\varpi }$*, while the average routing length in the phantom routing method is* ϒ *=* $\frac{2}{3}R$*|*R=hr.

**Proof.**  First, in the phantom routing method, there is only one routing path, and its length is the distance from the sink to the source node. Take any position within the network, which is x|x ∈ {0⋅⋅⋅ R} far from the sink. Then take a small segment of fan ring with angle dθ in a ring with width dx, as shown in the area Q in Figure 1 of [25]. The number of nodes in this area is xdθdxρ, and the sum of the distance from this area to the sink is xdθdxρ×x. The number of events generated in this area is xdθdxρ×xλ. Therefore, the distance from the source nodes in this area to the sink can be obtained as follows:

(25) $$\iint_{s}\rho \lambda x^{2}d\theta dx=\rho \lambda \int_{0}^{R}\int_{0}^{2\pi }x^{2}d\theta dx=\frac{2}{3}\rho \lambda \pi R^{3}$$

The total number of nodes is $\pi R^{2}\rho \lambda$, and the average distance from each node to sink is:

(26) $$ϒ=\frac{2}{3}\rho \lambda \pi R^{3}/\pi R^{2}\rho \lambda =\frac{2}{3}R$$

In this paper, the length of the RBCPSLP routing path is related to the number of generated source nodes. The more the number of source nodes, the more the routing branches, and thus the stronger the privacy preserving capability. In fact, the number of source nodes is related with energy harvesting capacity. The number of source nodes can be calculated by the following equation:

(27) $$g=\frac{M•\bar{E_{hrv}^{en}}}{n•\varpi \left(f(\varpi )+1\right)}$$

The routing length of each source node is (f(ϖ)+1), and the total routing length is:

(28) $$\Psi =g\left(f(\varpi )+1\right)=\frac{M•\bar{E_{hrv}^{en}}}{n•\varpi }=\frac{\pi R^{2}\rho •\bar{E_{hrv}^{en}}}{n•\varpi }$$

☐

**Figure 8:**
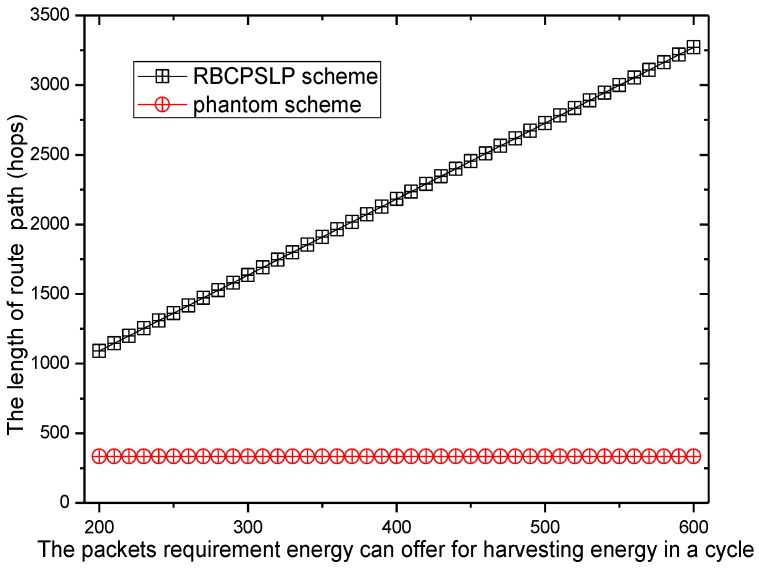
The length of route path in route schemes (with different $m_{1}$).

Figure 8 shows the comparison of routing lengths under different privacy preserving methods. The network scenario is as follows: network radius is R = 500 m, and the node density is 0.002/m^2^. The node’s energy harvested in an energy harvesting cycle (24 h) can provide nodes to send 200 to 600 data packets (denoted by $m_{1}$) with a data transmission cycle of 5 min (i.e., the number of time slots τ in an energy harvesting cycle is 24 × 60/5 = 288. It can be seen from Figure 8 that the routing length of the RBCPSLP scheme is 3.2 to 9.8 times the length of the phantom routing scheme, which means that the privacy preserving capacity is also increased by 3.2 to 9.8 times in the RBCPSLP scheme. Therefore, the RBCPSLP scheme proposed in this paper has better security.

**Figure 9:**
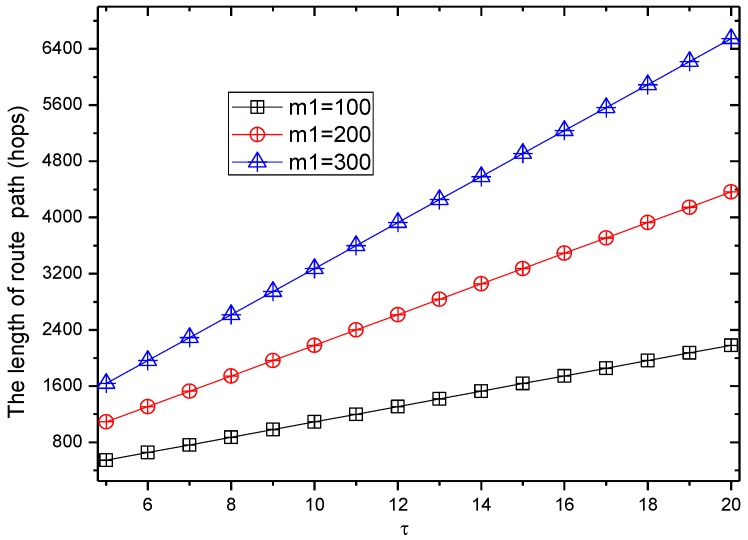
The ratio of route length of RBCPSLP vs. phantom (with different τ).

**Figure 10:**
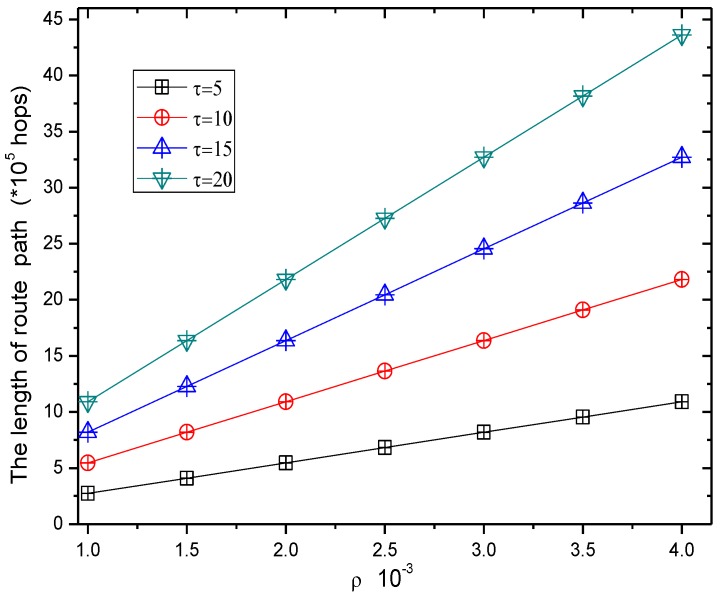
The ratio of route length of RBCPSLP vs. phantom (with different ρ).

Figure 9 shows the routing length in the RBCPSLP scheme at different data transmission frequencies. As can be seen from Figure 10, when the time slot τ of data transmission increases, it means that the number of data packets sent in one energy harvesting cycle becomes smaller under the fixed number of source nodes, and the energy consumption is reduced. However, the harvested energy does not change, and we can increase the number of source nodes in order to generate more data packets. Thus it is easier to confuse the adversary, and enhance the source location privacy preserving capability. Also, to increase the number of source nodes means to increase the length of the path. It can be seen from Figure 9, the greater the time slot τ, the larger the routing path length. Hence, the security is enhanced.

Figure 10 shows the routing length of the RBCPSLP scheme at different node densities. As can be seen from Figure 10, when the network node density increases, the number of network nodes increases as well, so that more energy can be collected. More energy can be used to create more false source nodes. Therefore, its routing length also increases, which means that privacy preserving capability is strengthened.

(2) Analysis of the capability to resisting the global attack.

The RBCPSLP scheme has the ability to resist global attacks. From the previous discussion, it can be seen that the generation of the source node in the RBCPSLP scheme is independently distributed in the whole network. Thus, even if an adversary with a global view detects that a node generates a data source, it cannot determine whether it is a real source node or not, and thus the RBCPSLP scheme has the capability to resist global attacks. This scheme proposed in the paper is capable of resisting the global attack but at only the price of resisting local attack.

### 5.3. Analysis of Energy Efficiency

The following analysis focuses on performance comparison between the proposed scheme in this paper and other privacy preserving schemes. The energy efficiency mainly refers to how efficiently the energy is used, that is, the percentage of efficient use of energy under the circumstance of no affecting the network lifetime. We have the following Theorem 3.

**Theorem** **3.** *In phantom routing scheme, if the network only allows for single event source, the energy utilization rate of the network will not exceed the following Equation (29),*

(29) $$\phi_{p}=\frac{n\varpi \left(\frac{2}{3}R\right)/r}{\pi R^{2}\rho •\bar{E_{hrv}^{en}}}$$

*Whereas the energy utilization rate in the RBCPSLP scheme can reach 100% in theory*.

**Proof.**  In the phantom routing scheme, the average route length of the event source is $\frac{2}{3}R$. The energy consumption of each event in a time slot is $\frac{2}{3r}R\varpi$, and here are n time slots in an energy harvesting cycle. Thus, the total energy consumption is $n\varpi \left(\frac{2}{3}R\right)/r$, and the energy harvested in an energy harvesting cycle of the entire network is $\pi R^{2}\rho •\bar{E_{hrv}^{en}}$.

In the RBCPSLP scheme, however, all the energy in theory can be consumed up. Therefore, the energy utilization rate is 100%. ☐

### 5.4. Analysis of Delay

**Theorem** **4.** *Considering each node is required to randomly select a time slot to send data with a fixed cycle* τ *as the source node, the cycle of energy harvesting is* T*. Suppose a node randomly select a time slot, and starts to send data from this time slot. The maximum number of time slots in which the node can keep sending data is* α*, and the duty cycle of each node in the network is* ατ*. The number of nodes in the same interference radius is* ξ*. The node will forward data once there is relay node, and the expected delay of the node is:*

(30) $$D=\sum_{i=0}^{T-2}i{\left(1-\alpha \tau \right)}^{i\xi }\left[1-{\left(1-\alpha \tau \right)}^{\xi }\right]$$

**Proof.**  The number of time slots that a node sends data is α. When a node is sending data, the other nodes cannot send data at the same time, which affects the nodes in the same communication range. If a node starts to send data, its delay is fixed in this cycle. The event of delay cannot be regarded as binomial distribution. The delay varies from 0 to ατ−1, and the probability density function of the delay in each slot is $P_{D}\{X=k\}={\left(1-\alpha \tau \right)}^{km}\left[1-{\left(1-\alpha \tau \right)}^{m}\right]$ where k=0,1,...,T−1. Thus, the expected delay of the node is:

$$D=\sum_{i=0}^{T-1}i{\left(1-\alpha \tau \right)}^{i\xi }\left[1-{\left(1-\alpha \tau \right)}^{\xi }\right]$$

☐

## 6. Experiments and Performance Analysis

We use the simulation tool Omnet++ [51] to simulate the effectiveness of the proposed scheme in this paper. The nodes of the simulation experiment are randomly distributed in a circular network. The nodes cannot be moved after deployment. The sink is located at the center of the sensor network. Each node in the network generates events with random probability λ. After the event occurs, the routing policy is used to route the event information to the sink. In the following experiment, unless stated otherwise, the length of the data packet is set to 10 bits. Other experimental parameters are shown in Table 2.

The data of collected energy are based on experimental data in Los Angeles (33° N, 118° W) from 1 August to 5 August in 1990, as shown in Table 3. The data records the light information every one hour in a whole day. The relation that the amount of the collected energy in the solar energy-driven sensor nodes is in a certain proportion with the solar radiation is taken into consideration.

Suppose that the final conversion rate from solar energy to the electricity is 10%, and the solar radiation changes randomly over time but also periodically. The solar radiation reaches the highest value at about 12 o’clock every day, and becomes 0 in the evening.

### 6.1. Comparison of Energy Efficiency

**Figure 11:**
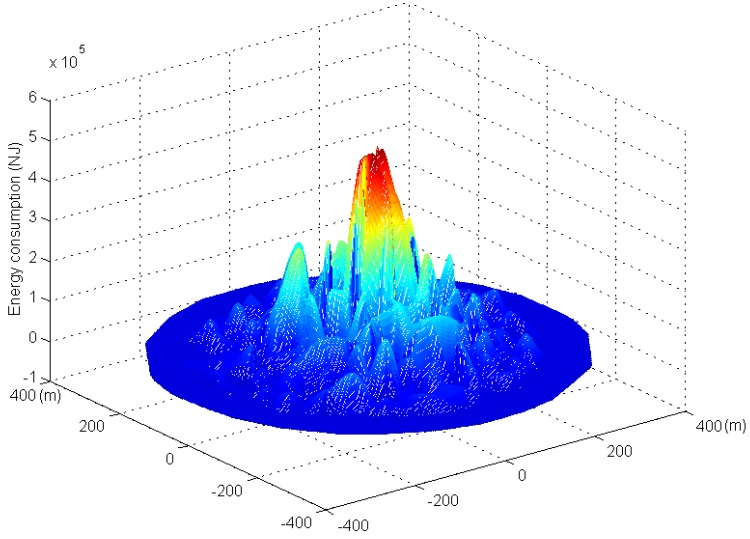
The network energy consumption in phantom scheme.

**Figure 12:**
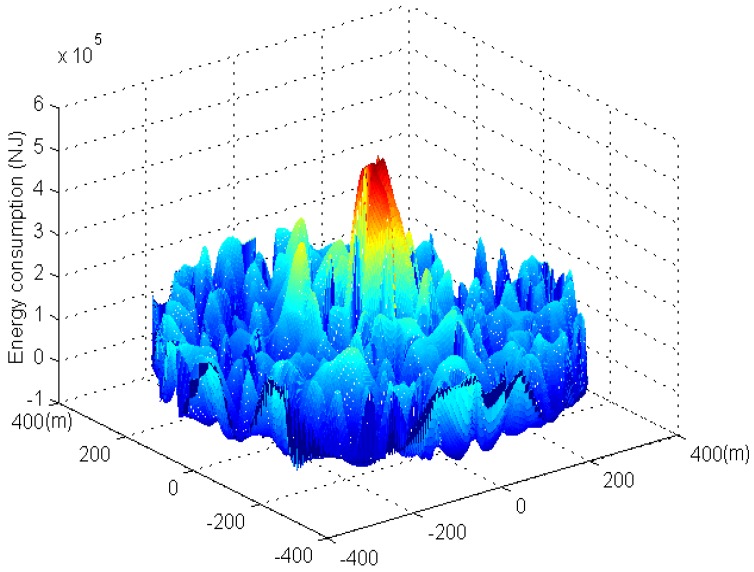
The energy consumption in RBCPSLP scheme.

Figure 11 and Figure 12 show the three-dimensional graphs of network energy consumption under the phantom routing scheme and the RBCPSLP scheme, respectively. It can be seen from Figure 11 that the energy consumption in the near sink area is high in the phantom routing scheme, while the energy consumption in the area far away from the sink is low. In the RBCPSLP scheme, due to the full use of the energy collected by the node, the energy consumption in the entire network is relatively balanced when a large of number of source events are created in the non-event area of the network, as shown in Figure 12. Therefore, the RBCPSLP scheme achieves high energy utilization rate by making full use of the network energy.

**Figure 13:**
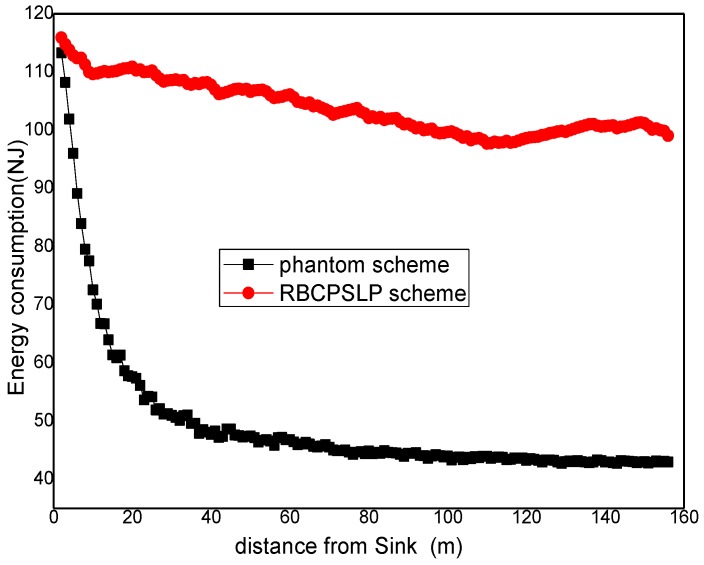
The energy consumption under difference scheme.

**Figure 14:**
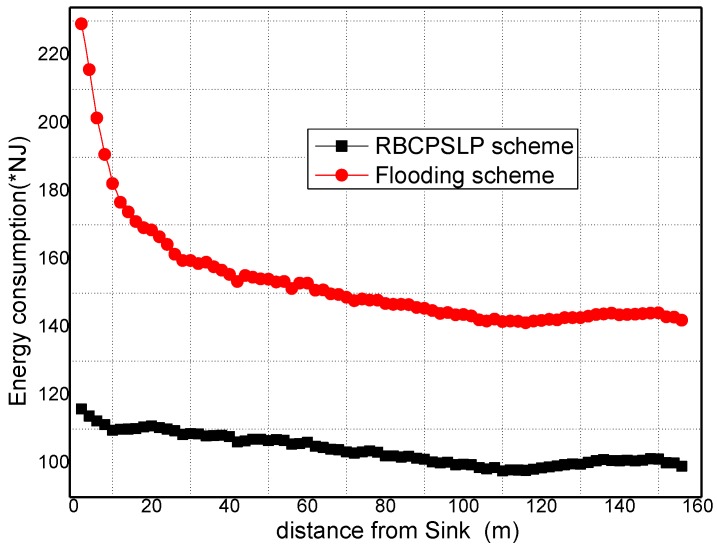
The energy consumption in flooding scheme and RBCPSLP scheme.

Figure 13 shows the energy consumption under different privacy preserving schemes. It can be seen from Figure 13 that the RBCPSLP scheme proposed in this paper basically obtains balanced energy consumption in different regions of the network (theoretically, the RBCPSLP scheme should be able to balance energy consumption in the whole network at equilibrium, however, nodes in the experiment are randomly distributed, and the events are also randomly generated, thus the energy consumption in practical experimenta is not very balanced. It may still occur that the energy consumption is high near the sink area but very low in the area far from the sink). In the phantom routing scheme, the closer to the sink the nodes are, the higher their energy consumption are. Thus, the energy consumption declines rapidly. Figure 14 shows the energy consumption in the flooding scheme and RBCPSLP scheme. It can be observed that the energy consumption of the flooding scheme is higher than the energy consumption in the RBCPSLP scheme, and the largest energy consumption of flooding scheme is also higher than the energy consumption in RBCPSLP scheme. The reason is that all nodes send data to the sink, thus the larger amount of data is sent to the sink, the higher energy consumption is in the hotspots.

**Figure 15:**
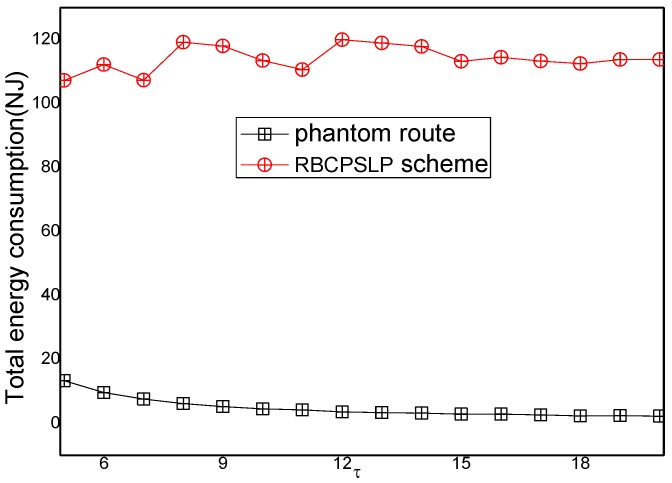
The total energy consumption (τ).

Figure 15 shows the comparison of the total energy consumption between the RBCPSLP scheme and the phantom scheme at different time slot τ. As can be seen from Figure 15, the total energy consumption in the RBCPSLP scheme is about 10 times greater than that in the phantom scheme. This is because the RBCPSLP scheme creates a number of source nodes, and all the source nodes are split into upside-down tree-like multi-branch routes through the aggregation routing algorithm, thus increasing their total energy consumption several times. However, this have no impact on the network lifetime since this energy belongs to the energy collected in the entire network. In fact, energy consumption is proportional with the length of the routing path, so a high total energy consumption means that the routing path of the RBCPSLP scheme is many times longer than that in the phantom scheme, which also means that the security of the RBCPSLP scheme is higher than that in the phantom scheme. It can also be seen from Figure 14 that in the RBCPSLP scheme, the total energy consumption of the network does not change with the data packet transmission cycle slot τ. The reason is that the RBCPSLP scheme determines the number of data packets to be sent based on the amount of collected energy. In the case of a fixed number of network nodes, the total energy that the network can collect is also determined, and its total energy consumption basically remains the same. In the phantom scheme, the transmission frequency of the data packet decreases with the increase of the time slot τ. Therefore, the total energy consumption in the phantom scheme is reduced.

**Figure 16:**
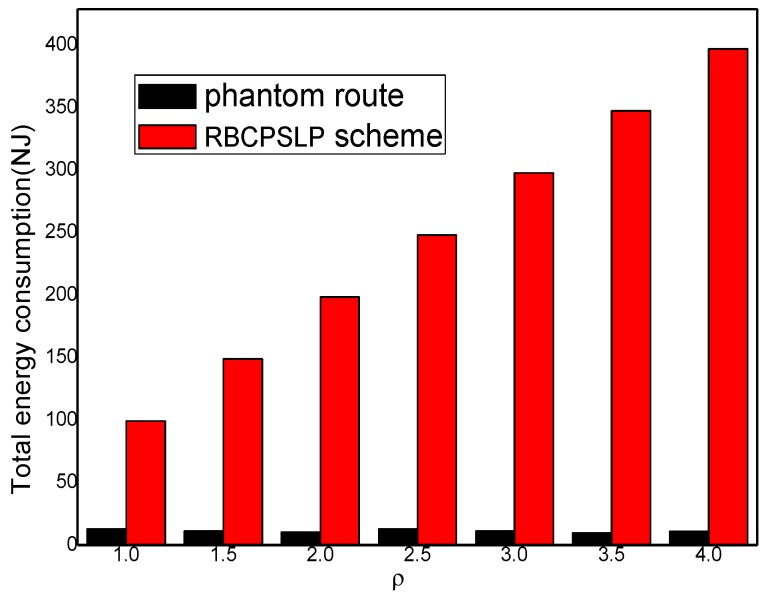
The total energy consumption (ρ).

Figure 16 shows the total energy consumption of the network at different node densities. It can be seen from Figure 16 that as the node density of the network increases, the total energy that can be collected in an energy harvesting cycle increases as well. The RBCPSLP scheme aims to make full use of the energy collected by the network. Therefore, the total energy consumption of the network increases as the node density raises.

### 6.2. Security Test

**Figure 17:**
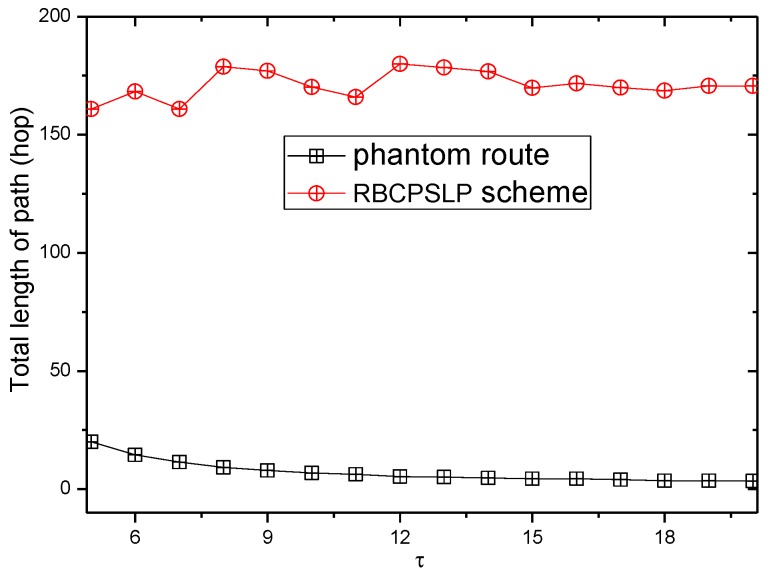
The total path length (τ).

**Figure 18:**
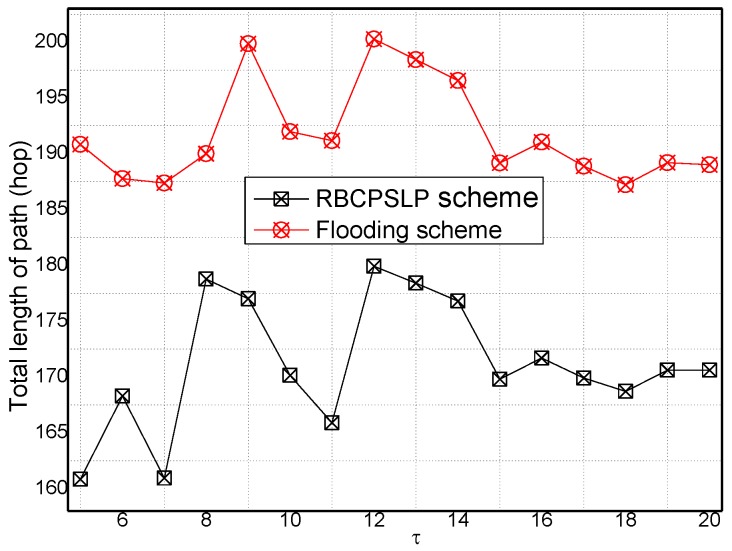
The total path length in Flooding scheme and RBCPSLP scheme (τ).

Figure 17 and Figure 18 show the total length of the routing path in the RBCPSLP routing scheme and phantom scheme, and the routing path in the RBCPSLP routing scheme and flooding scheme under different τ, respectively.

**Figure 19:**
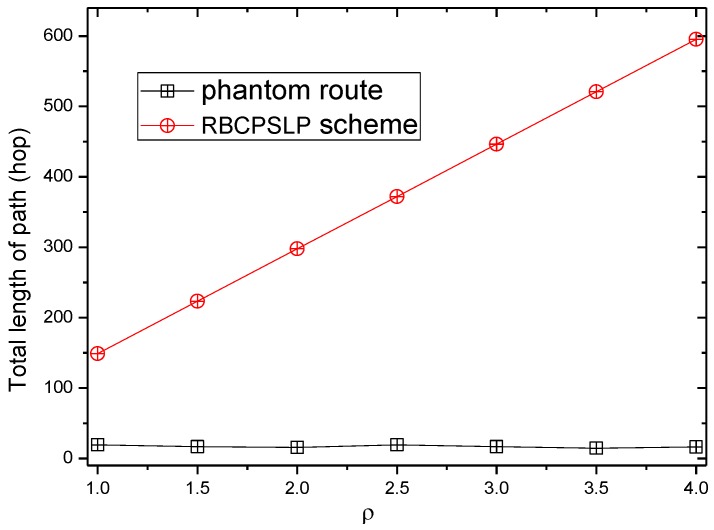
The total path length (ρ).

**Figure 20:**
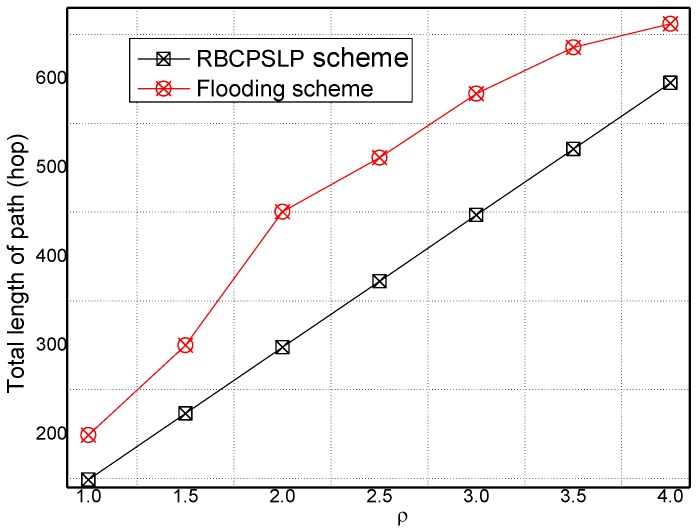
The total path length in Flooding scheme and RBCPSLP scheme (ρ).

Figure 19 and Figure 20 show the total length of the routing path in the RBCPSLP routing scheme, phantom scheme, and Flooding scheme under different ρ, respectively. As shown from the figures, the security of the flooding scheme is better than that of the other two. This is mainly because the flooding scheme does not take into account the entire network lifetime, and each node sends data to the sink. It can also be observed that although the flooding scheme has good security performance, it may have large energy consumption and low network lifetime. It can be seen from the experimental results that the routing length in the RBCPSLP scheme is 7.8 times longer than that in the phantom scheme. A longer routing length means a stronger security in privacy preserving capability. In the RBCPSLP scheme, a tree-like routing structure is used, in which each branch route in the tree may be a real branch route, or a false interference branch route. However, the adversary cannot distinguish it, so it can only tentatively attack the branch routing using the exhaustive search method. Since the path length is proportional to the security, a long routing path means the high security. The RBCPSLP scheme takes full advantage of the collected network energy, and achieves a total routing length that is 7.8 times the length of the phantom scheme one. Therefore, the RBCPSLP scheme has much higher security than the phantom scheme.

**Figure 21:**
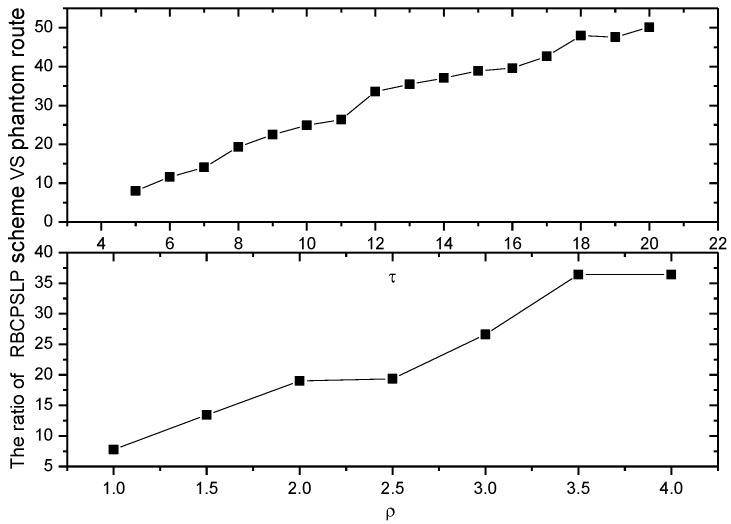
The ratio of phantom scheme vs. RBCPSLP scheme.

Figure 21 shows the ratio of the routing lengths between two different schemes. It can be seen that the total routing length in the RBCPSLP scheme is 7.8 times to 50 times the routing length of the phantom scheme.

**Figure 22:**
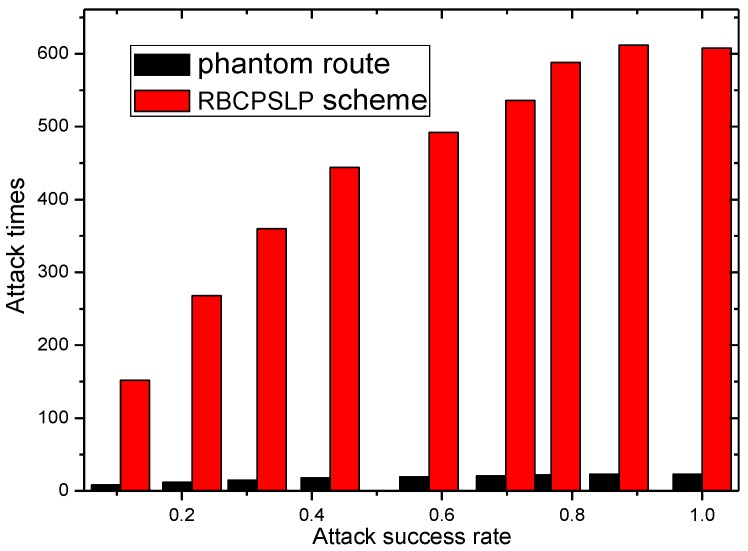
The attack times for certain attack success ratio under RBCPSLP scheme vs. phantom route.

The experimental scenario in Figure 21 is an experiment simulating the adversary’s reverse tracking to find the source node. The action that the adversary take is: when backtracking the source of the data packet, the adversary randomly selects a branch rout to attack once encountering branch routes. If the attack is not successful, then randomly selected another branch which has not been attacked until the attack reaches the pre-set attack hops. Moreover, we assume that the adversary is so intelligent that it spends no time to return to the new branch route to continue the next attack once the attack fails on the current branch route. Figure 22 shows the number of attacks required to achieve a certain probability of attack success under different schemes. It can be seen from the experimental results that RBCPSLP scheme makes full use of the energy collected by the network, and creates a lot of routing paths, which leads to the exhausted search for the adversary on a large number of branch routes. Also, the average number of attacks needed to achieve a certain probability of success becomes very large. Therefore, it is shown that the RBCPSLP scheme proposed in this paper has better security.

## 7. Conclusions

Wireless sensor networks have been widely used, and the rise of green networks enables the infinite lifetime of wireless sensor networks to become a reality, thus playing a great significant role. Privacy preservation will become a key factor in the future pervasive computing scenario for human use. Therefore, in this paper, we propose a source location privacy preserving scheme for the energy harvesting sensor networks. The RBCPSLP scheme predicts the energy collected in an ernegy harvesting cycle, and maximizes the privacy preservation by creating as many interference sources as possible. Then, the paper proposes a branch convergence-based source location privacy preserving strategy, so that the routes merge into a few routes before the arrival to the sink, thus eliminating the hotspots impacts the sensor network, so the energy utilization rate is improved significantly. Also, the privacy preserving strength in the netowrk is enhanced, therefore, the proposed work this paper has good practical significance.

## Figures and Tables

**Table 1:** Network parameters.

Parameter	Value
Threshold distance (*d*_0_) (m)	87
Sensing range *r_s_* (m)	15
*E_elec_* (nJ/bit)	50
*e_fs_* (pJ/bit/m^2^)	10
*e_amp_* (pJ/bit/m^4^)	0.0013
Initial energy (J)	0.5

**Table 2:** Network parameters.

Parameter	Value
Network radius (*R*) (m)	500
Protocols used	Shortest routing
Transmission radius of nodes (m)	50
Node density (/m^2^)	0.002
An energy harvesting cycle (hours)	24
A data transmission cycle (minutes)	5

**Table 3:** Value of radiation from 1 August to 5 August.

**Time (O’clock)**	**Solar Radiation Value (Wh/m^2^)**				
	8/1	8/2	8/3	8/4	8/5
1	0	0	0	0	0
2	0	0	0	0	0
3	0	0	0	0	0
4	0	0	0	0	0
5	0	0	0	0	0
6	0	0	0	0	0
7	27	20	21	19	21
8	134	57	71	74	99
9	428	126	194	172	293
10	637	426	228	272	615
11	808	696	365	338	790
12	929	848	607	375	905
13	994	948	853	613	969
14	988	967	882	577	973
15	928	837	765	592	911
16	808	581	659	697	761
17	636	319	547	478	578
18	431	328	280	394	383
19	214	142	189	189	163
20	47	27	36	37	32
21	0	0	0	0	0
22	0	0	0	0	0
23	0	0	0	0	0
24	0	0	0	0	0
